# Grouped Multilayer Practical Byzantine Fault Tolerance Algorithm: A Practical Byzantine Fault Tolerance Consensus Algorithm Optimized for Digital Asset Trading Scenarios

**DOI:** 10.3390/s23218903

**Published:** 2023-11-01

**Authors:** Jian Liu, Wenlong Feng, Mengxing Huang, Siling Feng, Yu Zhang

**Affiliations:** 1 School of Information and Communication Engineering, Hainan University, Haikou 570228, China; 21220854000156@hainanu.edu.cn (J.L.); fengsiling@hainanu.edu.cn (S.F.); 2 School of Computer Science and Technology, Hainan University, Haikou 570228, China; yuzhang2015@hainanu.edu.cn

**Keywords:** blockchain, digital asset, PBFT, packet consensus, Raft

## Abstract

Based on the practical Byzantine fault tolerance algorithm (PBFT), a grouped multilayer PBFT consensus algorithm (GM-PBFT) is proposed to be applied to digital asset transactions in view of the problems with excessive communication complexity and low consensus efficiency found in the current consensus mechanism for digital asset transactions. Firstly, the transaction nodes are grouped by type, and each group can handle different types of consensus requests at the same time, which improves the consensus efficiency as well as the accuracy of digital asset transactions. Second, the group develops techniques like validation, auditing, and re-election to enhance Byzantine fault tolerance by thwarting malicious node attacks. This supervisory mechanism is implemented through the Raft consensus algorithm. Finally, the consensus is stratified for the nodes in the group, and the consensus nodes in the upper layer recursively send consensus requests to the lower layer until the consensus request reaches the end layer to ensure the consistency of the block ledger in the group. Based on the results of the experiment, the approach may significantly outperform the PBFT consensus algorithm when it comes to accuracy, efficiency, and preserving the security and reliability of transactions in large-scale network node digital transaction situations.

## 1. Introduction

A new type of social ecosystem that can connect the physical and virtual worlds is flourishing as the multiple demands for virtual reality increase. However, with the rapid growth in data volume and data value, the evolving metaverse faces the needs and challenges of privacy, security, high synchronization, and low latency. Fortunately, the evolving blockchain can be used to fulfill the trusted constructs, data interactions, and computational needs of the metaverse [1]. Since the birth of Bitcoin [2], blockchain [3] has been used to build a new model for the establishment and transmission of trust in an environment of distrust with its characteristics of immutability, openness, and transparency. Blockchain applications have reached all aspects of society from the initial field of digital currencies to smart cities, cryptocurrencies, and healthcare [4,5,6]. For instance, the secure storage and sharing of healthcare data is an essential issue, and storing and sharing medical records on the blockchain offers a substitute to the traditional healthcare sector, which relies on wireless wearable devices and medical applications to provide healthcare services [7].

Blockchain-related technologies are used to facilitate asset trades in order to fulfill the demand for digital asset trading. To ensure the accuracy of transaction results, blockchain networks offer services by choosing nodes, building distributed ledgers, and choosing consensus procedures [8]. Many scholars have begun to conduct in-depth research on the application of digital asset scenarios.

For example, the present condition of the digital asset copyright market and issues with asset protection were examined in [9] along with the applicability of blockchain in digital copyright management and protection. Digital asset management, decentralized off-chain storage, and artificial intelligence model design were merged in [10] to create a more intelligent, GDPR-compliant framework for digital assets. To assist the intellectual property protection of digital content, safeguard the legitimate rights and interests of authors, and lower the cost of copyright protection, [11] developed a digital asset copyright depository platform using blockchain technology. To deal with the issue of central oversight based on a big platform and a huge database, as well as the challenge of collaborative development, [12] proposed the construction approach of a specialized disease repository based on a reliable information chain. Incorporating distributed ledgers, smart contracts, and asymmetric cryptography, [13] introduced a corporate information management system to increase the security and applicability of enterprise data assets.

In summary, scholars have analyzed the feasibility of blockchain technology to solve the problem of the current situation of digital assets. However, as market demands have changed, the performance of digital asset systems based on blockchain technology has become a constraint, such as consensus efficiency, communication complexity, and reliability. Aiming at the relatively little research on the consensus mechanism of digital asset transaction scenarios, a grouped multilayer consensus algorithm GM-PBFT is proposed based on the improvement of the PBFT and Raft algorithm [14]. The principal work of this paper is as follows.

(1) A grouped multilayer-based PBFT consensus algorithm GM-PBFT is proposed to optimize and be improved by node grouping strategy, supervisory strategy, and grouped multilayer consensus process for the PBFT algorithm.
(2) The GM-PBFT algorithm is evaluated experimentally and compared with the PBFT algorithm in terms of communication overhead, consensus delay, and throughput.
(3) The node grouping strategy of GM-PBFT and the supervision strategy of supervising nodes are discussed to prove the security of the algorithm and to demonstrate the effectiveness of the algorithm.

## 2. Related Work

The PBFT consensus algorithm [15,16] was improved by Castro based on the BFT algorithm for solving the consensus problem of distributed systems in a blockchain environment. The PBFT algorithm tolerates Byzantine nodes and reduces the communication complexity from $O\left(n^{3}\right)$ to $O\left(n^{2}\right)$. Through three stages, the PBFT algorithm ensures the consistency of the consensus node confirmation message by passing the signature verification to judge the consensus node message. With the continuous addition of new nodes in the transaction link, which has problems such as high communication complexity, poor scalability, and repeated election of malicious nodes to the main node, scholars have started to improve the PBFT consensus algorithm. In general terms, there are five various kinds of improvement strategies.

(1) Adopting digital signature technology. Sheng [17] et al., in order to reduce the probability of view change, evaluated the node trust by the efficiency of processing transactions between nodes, selected nodes with high trust to join the consensus group, and used group signature and mutual supervision strategies to enhance the algorithm stability. Fan [18] introduced the S-PBFT consensus algorithm, which replaces the digital signature in the consensus process with a short-term signature, and the dynamic changes of the nodes are controlled by the key distribution mechanism of the blockchain, which increases the dynamics of the system and reduces the latency and resource consumption.
(2) Adopting a representative committee consensus instead of an all-node consensus. Liu [19] et al. utilized the integral grouping mechanism to select nodes with high trust to form a consensus group, use the consensus group instead of all the nodes to carry out consensus, and assign the voting value to the nodes of the consensus group to reduce the amount of communication in the consensus process. This solution addressed the issue of random selection of master nodes and poor expandability.
(3) Adopting a simplified three-stage protocol process. Gan [20] et al. introduced an EPBFT consensus algorithm that fades the master node model, which removes the commit phase from the three-stage process in PBFT and reduces the number of communication levels for consensus. Liu [21] et al. proposed a multi-party authentication digital asset platform based on a threshold ECDSA algorithmic security protocol to allow participants to confirm the authenticity of the assets, embedding a verifiable Byzantine fault tolerance (VBFT) mechanism, randomly selecting consensus nodes, and improving the security of the nodes.
(4) Adopting a credibility voting mechanism. Tu [22] et al. sought to solve the problem of inefficiency caused by the master node being a Byzantine node and the excessive number of nodes in the cluster in the PBFT consensus algorithm. The PBFT algorithm was improved using the reputation voting method to improve the consensus efficiency. Gu [23] et al. utilized machine learning for node anomaly detection and used the result of anomaly detection as a factor for evaluating the reputation value. Since the master node can generate blocks, random numbers are added to the reputation model to prevent centralization and ensure the fairness of the master node election.
(5) Adopting layer structure. Feng [24] et al. designed a dynamic hierarchical approach of agent nodes where PBFT consensus operates in a specified region to reduce the complexity of the whole system. Li [25] et al. grouped nodes into different layers and restricted intra-group communication to reduce the communication complexity due to frequent inter-node communication and poor scalability of the node PBFT mechanism. Duan [26] et al. established the trust degree based on the operational status of the nodes and elected trusted agent nodes for the local and global consensus process twice, thus reducing the number of message broadcasts. In order to establish a PBFT-based blockchain system in an instance involving a large-scale network, Luo [27] presented an ideal two-tier PBFT consensus that reduces storage overhead while limiting the storage overhead within a group.

The analysis of the improved PBFT consensus algorithm reveals that when the PBFT algorithm sends a consensus request in the blockchain network, the system nodes need to agree on he same transaction request at the same time. However, as more nodes join the blockchain network, it becomes more difficult to solve the scalability and adaptation to large-scale network issues at their core by simply enhancing the PBFT algorithm. As a result, the system’s consensus efficiency declines, its reliability deteriorates, and its communication overhead grows linearly. As an effect of performing hierarchical multigroup consensus utilizing the GM-PBFT method, the problem can be solved in this study by employing the grouped multilayer PBFT consensus algorithm.

## 3. GM-PBFT Algorithm Design

Despite possessing more communication complexity, the PBFT algorithm provides greater Byzantine fault tolerance. The Raft algorithm consensus is efficient and has excellent scaling performance but has no Byzantine fault tolerance. With the combination of the Byzantine fault-tolerant feature of the PBFT algorithm and the efficient consensus of the Raft algorithm, a multi-component layer consensus algorithm GM-PBFT is proposed.

The GM-PBFT algorithm consists of three parts: node grouping policy, supervisory policy, and grouping multilayer consensus. The nodes are accurately classified into different consensus groups by their professional background and application experience attributes. The pheromone concentration of the ant colony optimization algorithm [28] is employed within the group to find the optimal nodes within the group to ensure the accurate division of the sample.

**Figure 1:**
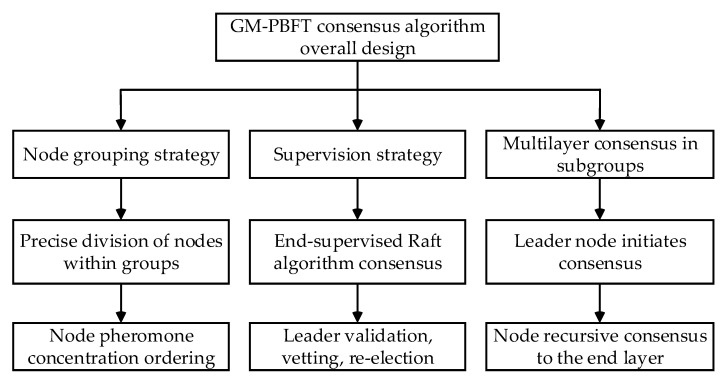
Overall design of the GM-PBFT algorithm.

The node with the highest pheromone concentration acts as a supervisory node to supervise the leader of the consensus of the Raft algorithm at the end layer and guarantees the reliability of the consensus through validation, auditing, and reselection mechanisms. The grouped multilayer consensus is performed by the leader node through the PBFT algorithm for hierarchical recursive consensus to reduce the communication complexity of the system. The overall design of the GM-PBFT algorithm is shown in Figure 1.

**Figure 2:**
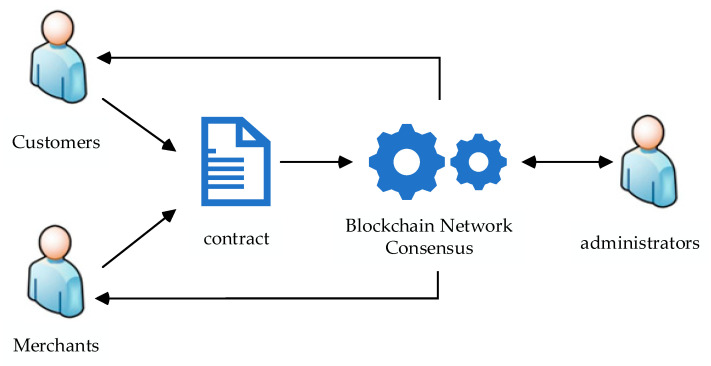
Digital asset trading scenario.

Assuming a digital asset trading scenario as shown in Figure 2, the merchant publishes relevant information services, and the purchaser queries for information according to their demands. The ledger is created in accordance with the specifics of the transaction when the buyer and seller successfully arrange a trade for e-book rights. For instance, the information such as the price of the item, the age of the item, and the accounts of both parties.

A transaction consensus request is generated through the contract and sent to the blockchain network. The blockchain network will generate a consensus on the transaction and return the result to both parties of the transaction. However, the communication complexity during consensus is too high, which will lead to a higher probability of node error as well as lower consensus efficiency. The administrator node is adopted to supervise the consensus phase to secure the consensus phase.

When the commodity consensus group receives the client request from the main node, it will initiate consensus on the transaction content within the group and send the result to the main node with its consensus success. The main node sorts and packages the transactions with successful consensus to generate blocks and sends the blocks to each transaction consensus group to guarantee the consistency of the block ledger of each node.

### 3.1. Node Grouping Strategy

The administrator will oversee the consensus process while the merchant and the customer will negotiate the transaction’s terms. A total of three parties are involved in the digital asset transaction process: the administrator, the merchant, and the customer. The node can handle transactions normally, or it can be a master node that manages transaction consensus, or it can be a supervisory node before the consensus stage, where each node can be categorized by transaction type.

Blockchain networks have similarities with the traveler’s problem, so it is feasible to use the ant colony algorithm to solve the node pheromone concentration in digital asset transactions. The ant colony method and node contact range are assessed for integration based on the properties of blockchain theory, as well as the procedure and mechanism of digital asset transactions. The nodes in the group are processed by an ant colony optimization algorithm to find the highest pheromone node in the group to act as the supervisory node. Higher pheromone concentration indicates that the node is more closely connected to other nodes in the group and has a higher trust degree.

The nodes in the group are numbered, the frequency of contact between nodes corresponds to the pheromone concentration, and the probability of no contact between nodes corresponds to the pheromone volatilization coefficient. The search strategy of the ant colony optimization algorithm is modified, and a node within the group is randomly selected as the starting point for matching. In the ant colony algorithm, $P_{m}\left(t\right)$ denotes that at the moment t, the artificial ant m starts at node i, then the probability of matching with node j is:

(1) $$P_{m}\left(t\right)=\left\{\begin{array}{c} \arg\max\{[\tau_{ij}\left(t\right)\cdot \left[\eta_{ij}\left(t\right)\right]\},j\in J_{m}\left(i\right)\\ \;0\;,otherwise \end{array}\right.$$

In the formula, $\tau_{ij}\left(t\right)$ is the pheromone concentration between node i and node j, and $J_{m}\left(i\right)$ is the set of all nodes in the group. $\eta \left(i,j\right)$ is the heuristic factor affecting the nodes, and the heuristic function expression is as follows:

(2) $$\eta_{ij}\left(t\right)=0.5\left(x_{i}+y_{j}\right)$$

In the formula, $x_{i}$ and $y_{j}$ are the difficulty of negotiation between the merchant and the customer; a harder negotiation implies a smaller $\eta_{ij}\left(t\right)$. C is the number of valid matches in the current node matching.

(3) $$\Delta \tau_{ij}\left(t\right)=\left\{\begin{array}{c} C\;,\left(i,j\right)\in n\\ 0\;,otherwise \end{array}\right.$$

$\Delta \tau_{ij}\left(t\right)$ is the pheromone increment on $\left(i,j\right)\;$ that ant m explores effective matches to stay on $\left(i,j\right)$, ρ is the pheromone volatilization coefficient in the system, and 1−ρ is the pheromone residual coefficient, $\rho \in \left(0,1\right)$. Then, the amount of pheromone that node i matches with node j at moment t+n is updated as follows:

(4) $$\tau_{ij}\left(t+n\right)=\left(1-\rho \right)\tau_{ij}\left(t\right)+\rho \Delta \tau_{ij}\left(t\right)$$

All nodes in the group need to be updated with pheromones in order to comply with the requirements of multi-objective transaction matching. The improved ant colony algorithm searches for the optimal solution between the nodes in each group through pheromone concentration. The node corresponding to the optimal solution can provide a supervisory node for the group consensus in the later stage, which improves the efficiency of the transaction matching between the two sides.

### 3.2. Supervision Strategy

The nodes in the k layer form a committee using the Raft consensus algorithm with three roles: leader, follower, and candidate. The leader decides whether to store new log messages and has the power to send data or not. Raft consensus node roles are able to change between the leader, follower, and candidate as conditions persist.

When a node joins the system, the committee determines whether there is a leader and if so, the node’s role is that of a follower. The system will only ever have one leader at any given time. In the event that there is no communication between the leader and other nodes, the committee elects the leader once more, and the follower becomes a candidate. The candidate will send a voting request to other nodes to apply to become the new leader. This candidate will be elected as the leader if it receives confirmation messages from more than half of the nodes within the time period, and the other nodes will be converted from candidates to followers.

In the log replication stage, all transactions are packaged by the leader to generate blocks and broadcast messages to the followers. The leader in the Raft algorithm is the strong leader; when more than half of the followers’ replies are received, the leader will send an acknowledgment message to all the nodes, and the block will be submitted to the chain. If the leader node is a malevolent node and sends false messages to the followers, it has an impact on the consistency of the consensus process. This paper changes this situation by introducing supervisory nodes.

Supervisory nodes monitor the behavior of leaders in the group; in order to ensure that the supervision is effective, the supervisory nodes do not participate in the election of the leader and only participate in the group as a follower of the Raft consensus. When the supervisory node receives the leader log message, it needs to verify the signature for comparison, and the malice-free node will broadcast the message within the group. Supervisory nodes are regulated in three steps for leaders: validation, vetting, and re-election.

(1) Verification: When the leader transmits a log message to a follower during the group Raft consensus phase, it is required that they both sign the message. The supervisor node collects the log messages sent by the leader to the followers and compares and verifies them. When the supervisory node successfully completes the verification, it demonstrates the consistency of the log messages transmitted to the followers. The supervisory node will examine the leader if he or she posts a false message.
(2) Auditing: The supervising node sends a verification request message to the follower and the follower sends a log message to the supervising node to review the leader. The supervisory node finds that the follower receives the log message inconsistently by comparison, and then it decides that the leader node is a malicious Byzantine node.
(3) Re-election: The supervising node requests the committee to remove the leader’s status and resume the leader election process after concluding that the leader is an evil Byzantine node.

Supervisory nodes can decrease the possibility of leader misbehavior through verification, auditing, and re-election mechanisms. Raft consensus in the group introduces the monitoring method; the supervision node can spot the leader’s sinister behavior in time and start the process of electing a new leader to guarantee the safety of the consensus stage.

### 3.3. Group Multilayer Consensus

Based on the type of asset transaction, the consensus nodes are divided into various groups, such as the commodities transaction consensus group, the securities transaction consensus group, the artwork transaction consensus group, and the cryptocurrency transaction consensus group. Each transaction consensus group is responsible for validating the relevant transaction affairs and communicating the validation results to the consensus group’s main node. The individual transaction consensus groups sort and package the successfully validated transactions and generate blocks.

To ensure the consistency of the groups’ ledgers, the main node transmits the created block to each transaction consensus group at the end. The GM-PBFT is applied to the upper node PBFT consensus pseudocode, which is designed as follows (Algorithm 1):
**Algorithm 1:** GM-PBFT upper node consensus pseudocode.Input: transaction request  v is the view number, n is the sort number, and d is a summary of the client request message m. Output: consensus to k layer 1. the main node sorts the messages received from the client; 2. begin initializing the first layer a = 0; 3. for a = 0 to k do 4.  send a request message to the next level node; 5.  while request valid = true do 6.    send pre-prepare message; 7.    number = 1; 8.    if prepare valid = true then 9.     number = number + 1; 10.      if number > 2f + 1 then 11.       send commit certificate; 12.     end 13.     if commit valid = true then 14.      send reply client; 15.     end 16.    end 17.   end while 18.   a = a + 1; 19. end for 20. send consensus request to k layer; 21. end

Taking the commodity group as an example, node A22 is the leader, nodes A31, A32, A33 and G1 are the followers, and node G1 is also the supervising node. Node G1 is responsible for supervising node A22 to prevent the leader from sending false messages. This is so that the group’s consensus cannot remain consistent once the leader transforms into a resentful Byzantine node and begins to send messages to the other nodes.

The three steps of the consensus process are the pre-preparation stage, the preparation stage, and the submission stage. The top layer of nodes employs the PBFT algorithm consensus. In the pre-preparation stage, a client message is broadcast by the main node S to the slave nodes A11, A21, A22, A23, and A24. In the preparation stage, the preparation messages are broadcast by the slave nodes A11, A21, A22, A23, and A24 after receiving the pre-preparation messages sent by the main node S. In the commit stage, the main node S broadcasts an acknowledgment message to all consensus nodes and receives acknowledgment messages from other nodes. When it receives no less than 2f+1 valid confirmation messages, it is determined that the node has completed the three stages of consensus. The consensus result is delivered to the next layer and the PBFT algorithm consensus is continued until the K layer is reached.

The Raft algorithm consensus is used by the layer k nodes, and A22 packages the transaction to generate a block and broadcasts it to A31, A32, A33, and G4. When a majority of the follower replies are received for confirmation, A22 sends the validation confirmation to all the nodes.

**Figure 3:**
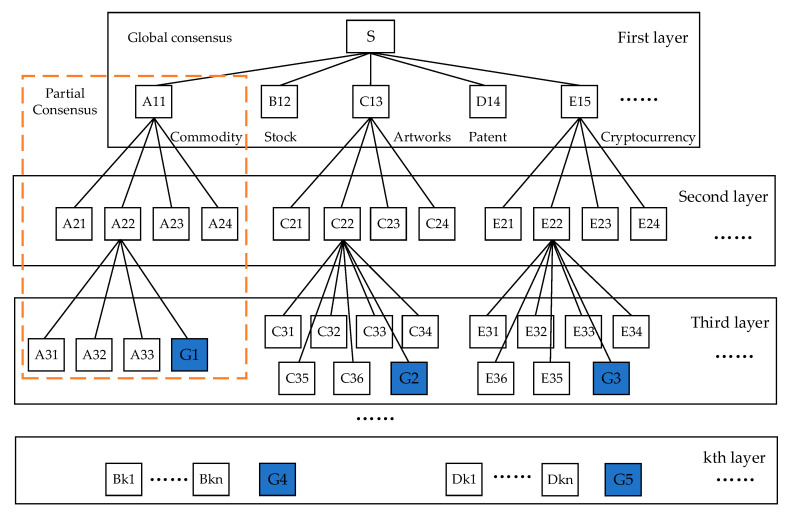
Grouping Multilayer Consensus Model.

Since the leader might turn out to be a malicious node, a supervisory policy mechanism is used to regulate the leader’s behavior in sending messages. Figure 3 shows a multilayer consensus model based on node attribute grouping.

### 3.4. GM-PBFT Consensus Process

All nodes can be classified via preprocessing based on node attributes prior to the consensus phase of the group of nodes. The enhanced ant colony algorithm sorts the nodes into groups according to their pheromone concentrations. The node in the group with the highest pheromone concentration is the supervisory node, while the other nodes are regular nodes.

The first layer master node initiates a PBFT consensus request to the second layer node, and the result is sent to the first layer master node and delivered to the client after successful consensus. The second layer master node initiates another submission request to the next layer, which is executed recursively until the kth layer node. The kth layer node forms a committee with the leader node of the previous layer, and the leader and the kth layer node perform the Raft algorithm consensus. For the sake of the consistency of the messages provided by the leader, the kth layer also adds supervisory nodes.

**Figure 4:**
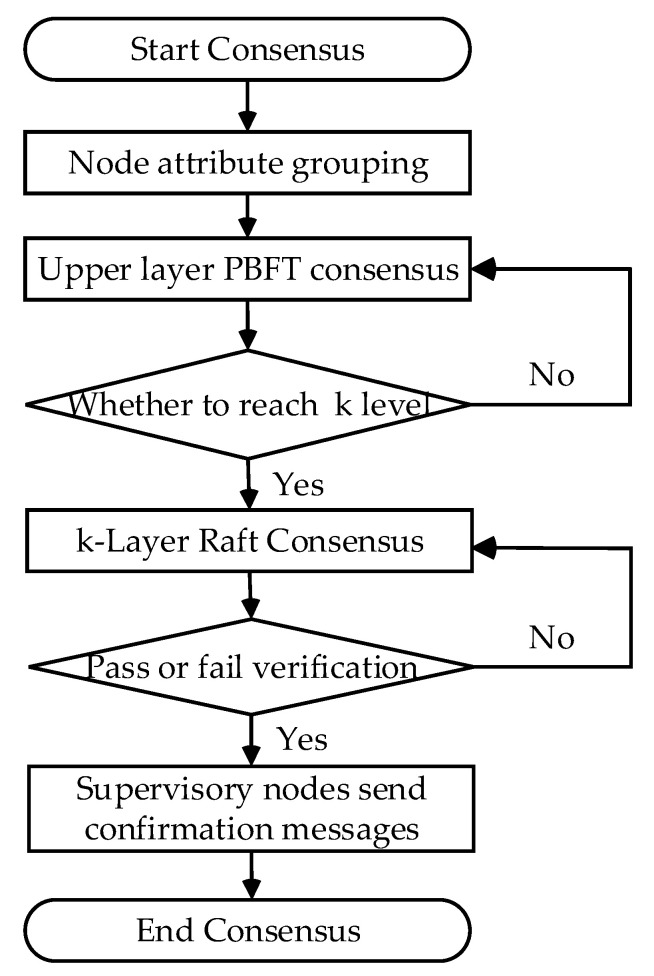
GM-PBFT consensus process.

The supervising node collects messages from other nodes and validates the leader message after receiving it to ensure its accuracy. The log message is then broadcast to other nodes after passing to complete the verification within the group. The overall process is shown in Figure 4.

Accessing the first layer of the blockchain network, the main node and the second layer nodes form a consensus group. When the main node receives a client proposal, it propagates the message to the $X_{2\;1}$, $X_{\;2\;2}$, and $X_{2\;3}$ nodes. The slave node receives the pre-prepared message from the main node and starts to verify the legitimacy of the consensus node. It propagates a vote message with its own vote to the whole network if the verification is passed and writes it in the message log. The master node propagates an acknowledgment message with its own vote value to the whole network and collects acknowledgment messages from other nodes.

**Figure 5:**
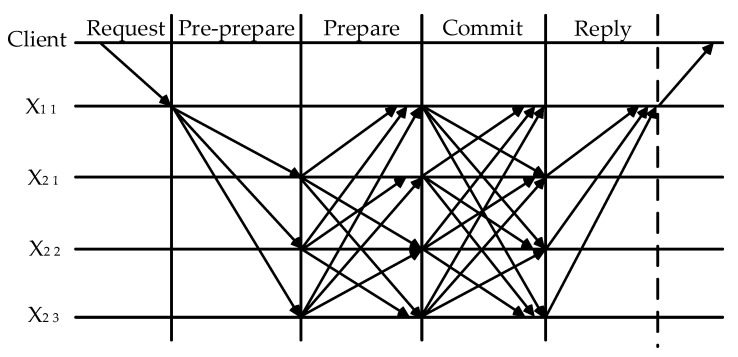
First layer PBFT consensus process.

If the consensus node receives no less than 2f+1 confirmation messages, it initiates the PBFT consensus request with the consensus result to the next layer nodes in the same group. In this context, f is the number of Byzantine nodes in the group, $X_{a\;b}$ indicates the bth node of the ath layer, and the first layer PBFT consensus flow is shown in Figure 5.

**Figure 6:**
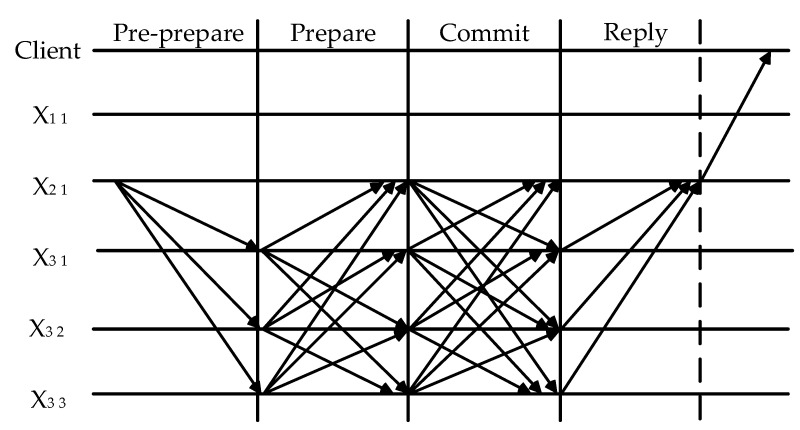
Second layer PBFT consensus process.

When the leader node $X_{2\;1}$ receives the proposal, it propagates the message to the nodes $X_{3\;1}$, $\;X_{\;3\;2}$, and $X_{3\;3}.$ The second layer node $X_{2\;1}$ sends the message to all the nodes in the third layer and each node will receive the relevant information such as node signature, view number, etc. After the pre-preparation phase receives the message, the preparation phase generates a certificate and the submission phase will wait for at least 2f+1 nodes’ same node signature, view number, and other related information. Receiving the certificate of the submission phase, the request message is sent to the leader node and passed to the client. The leader node of the third layer makes a submission to the same group of lower layer nodes to launch the PBFT consensus request again, and there is a recursive consensus to the last layer. The second layer PBFT consensus process is shown in Figure 6.

The upper layer PBFT consensus node will take over as the Raft consensus leader once the consensus request reaches layer k. Within the group, the leader conveys a message to all followers after collecting all transactions into a block and broadcasting it to the group as a whole. The block is added to the chain when the leader sends an acknowledgment message to every node and more than half of the followers have responded.

**Figure 7:**
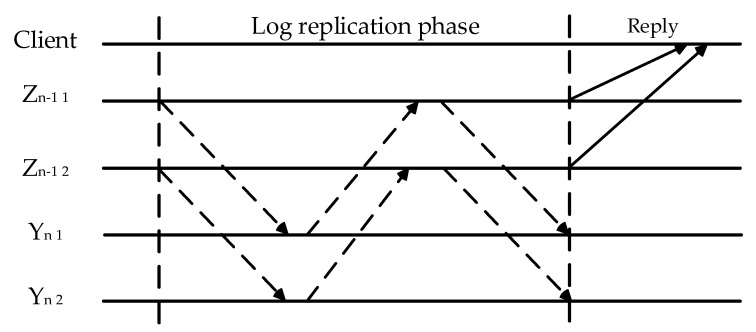
K Layer Raft Consensus Process.

If the node reply threshold is not reached, the supervising node triggers the leader reselection mechanism to perform the group Raft consensus again. In case the leader appears as a Byzantine node, the supervisory strategy will be used. $Y_{a\;b}$ represents group b in layer a, and $Z_{a\;b}$ represents the leader node of group b in layer a. The K layer Raft consensus process flowchart is shown in Figure 7.

## 4. Analysis of the Experiment

With the GM-PBFT algorithm as a consensus algorithm for federated chains, it is necessary to work with a system with a small number of malicious Byzantine nodes by simulating the blockchain network and experimentally recording the transaction data latency and throughput during the consensus process. In this section, four aspects of communication overhead, delay, throughput, and fault tolerance are analyzed to compare the results of pre- and post-optimization to experimentally demonstrate the advantages of the GM-PBFT consensus algorithm.

### 4.1. Experimental Environment

First, configure the Ubuntu operating system and virtual machine on the PC and allocate memory and disk size for the virtual machine. Next, install the Docker environment on the Ubuntu system, then download the Fabric version 2.2 source code and extract it to install and configure it. Run the byfn.sh script to start the Fabric network by going to ~/gowork/go/src/github.com/hyperledger/fabric/scripts/fabricsamples/first-network/trade.

Using the go language to write auto-executable code encapsulated into a corresponding smart contract such as trade.go, after starting the cli and nodes, execute the peer chaincode, and install -n basic -v 1.0-p github.com/chaincode/trade command to install trade.go in the docker container. Simultaneously with the initial end page code, the backend logic code is packaged, uploaded, and deployed to the server. The remote client then accesses the digital assets to trade the system and run latency and throughput performance tests.

### 4.2. Communications Overhead Analysis

The PBFT consensus mechanism requires two-two nodes to communicate with a traffic of $O\left(N^{2}\right)$, and N is the number of nodes. The Raft consensus mechanism has a traffic of O(N), and by grouping the network, the traffic of GM-PBFT decreases from $O\left(N^{2}\right)$ to $O\left(N/k\right)+O\left(k^{2}\right)$, abd k is the number of partitioned groups, compared to the PBFT consensus mechanism.

The number of communication times between nodes in the blockchain network represents the communication complexity; a lower number of times means the communication complexity is lower, and the scalability of the system is better. The number of communication times required for the consensus of the GM-PBFT algorithm is contrasted with that of the traditional PBFT algorithm. The number of node layers of the blockchain network is k (k ≥ 3), the number of nodes in each layer is n (n ≥ 3), and the total number of nodes is N.

As the blockchain network adopts the PBFT algorithm for consensus, the main node sends a pre-prepared message to the slave nodes with a communication count of N−1. The slave nodes send a prepared message to the rest of the nodes with a communication count of ${\left(N-1\right)}^{2}$. All the nodes will send acknowledgment messages to other nodes for $N*\left(N-1\right).$ The consensus node will send a reply message to the client, and the number of the communication is N. Then the number of PBFT consensus communications is:

(5) $$Q_{1}=N-1+{\left(N-1\right)}^{2}+N*\left(N-1\right)+N=2N^{2}-N\;$$

As the blockchain network adopts the GM-PBFT algorithm for consensus, in the case of a trademark consensus group, the number of communications for the first k−1 layers to perform PBFT consensus is $\left(k-1\right)*\left(2n^{2}-n\right)$. k layers use the Raft algorithm for consensus, and the leader broadcasts blocks to the followers as well as the supervisory node, with a number of communications of n. The supervisory node collects the follower logs, with a number of communications of n−1. The submit phase supervisory node sends a confirmation message to followers and validation message to leaders with a communication count of $2\left(n-1\right)$. GM-PBFT consensus communication count is:

(6) $$Q_{2}=\left(k-1\right)*\left(2n^{2}-n\right)+n+\left(n-1\right)+2\left(n-1\right)=\left(2k-2\right)n^{2}+\left(5-k\right)n-3$$

**Figure 8:**
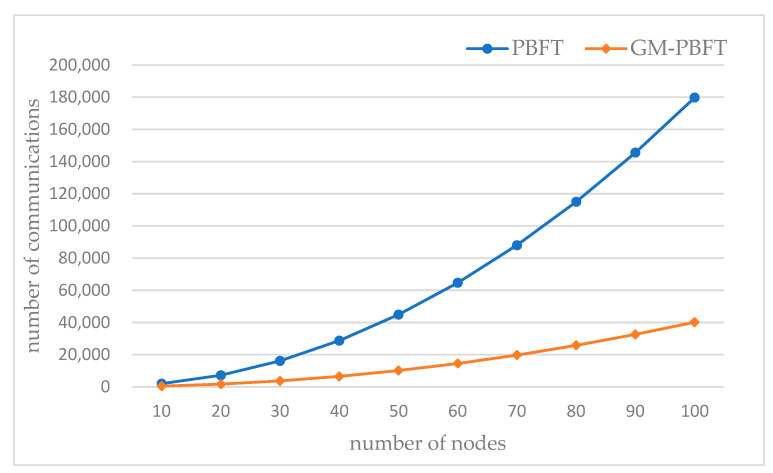
Comparison of the impact of node numbers on communication overhead.

When k=3, the communication overhead required for a single consensus of the GM-PBFT consensus algorithm is analyzed and compared with the traditional PBFT algorithm. Figure 8 shows the comparison of the effect of the number of nodes on the communication overhead.

From the figure, it is known that the communication complexity of both the GM-PBFT and the PBFT consensus algorithms increase with the increase in the number of nodes in the same network situation. As the number of nodes increases, more neighboring nodes need to communicate, thus increasing the communication complexity. When the number of nodes is the same, the communication overhead of the GM-PBFT consensus algorithm is lower than that of the PBFT consensus algorithm. The PBFT consensus algorithm can lead to a significant increase in the performance of the Byzantine fault-tolerant consensus, but the communication complexity is still huge.

The GM-PBFT consensus algorithm effectively reduces the number of consensus communications when the number of nodes increases. When the number of nodes at each layer in the system is 30, the number of GM-PBFT communications is reduced significantly compared to the PBFT algorithm. The beneficial effect of lower communication overhead increases as the system’s nodes are superior to 50.

### 4.3. Delay Analysis

Delay is an essential property indicator that measures the efficiency of the consensus algorithm as well as the performance of the blockchain network. It refers to the time it takes from the client initiating the request to the block successfully being uploaded to the chain. If the transaction experiments take less time, it means the consensus is reached more quickly and the system performance of the blockchain system is better.

DelayTime represents the time delay, $Time_{req}$ represents the moment of initiating the transaction request, and $Time_{reply}$ represents the moment of completing the transaction confirmation. The consensus delay calculation formula is shown below:

(7) $$DelayTime=Time_{reply}-Time_{req}$$

**Figure 9:**
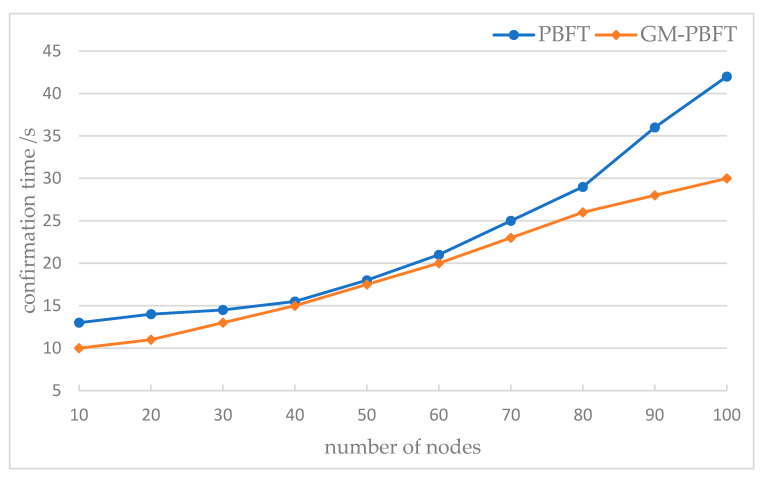
Comparison of the Influence of Node Number Graph on Consensus Delay.

The average value of the data from several experiments is used as the end result to test the consensus latency. This section of the experiment tests the GM-PBFT algorithm and PBFT algorithm for different numbers of nodes in the system network when the number of nodes in the system network is 10 to 100, respectively, and compares and analyzes the change in transaction delay. The time interval of each transaction message generation and chaining is calculated, which is converted to transaction delay, and some of the selected data are plotted as folds. Figure 9 shows the comparison of the impact of the number of nodes on the consensus delay.

Based on the graph, the consensus delays of the two methods increase gradually as the number of nodes rises; however, the GM-PBFT algorithm’s delay increases more gradually while the PBFT algorithm’s delay increases more dramatically. The grouping of several layers during the consensus process changes when there are more than 50 nodes, and the increased consensus performance of the GM-PBFT algorithm has some bearing on the consensus time.

The single-layer PBFT consensus method and the GM-PBFT algorithm described in this paper generally perform well in terms of latency as the number of nodes increases, with GM-PBFT having lower latency than the standard PBFT for the same number of nodes. Therefore, the GM-PBFT method can significantly reduce latency when used with large nodes.

### 4.4. Throughput Analysis

In blockchain systems, throughput refers to the number of transactions per second (TPS) processed by the system, reflecting the system’s ability to process transaction information. A high throughput of transaction data implies that the consensus algorithm used has a good performance. Δt is the time to complete the transaction consensus, and Transactions is the total number of transactions processed during the time to complete the transaction consensus. The throughput is calculated by the formula:

(8) TPS=Transactions/Δt

**Figure 10:**
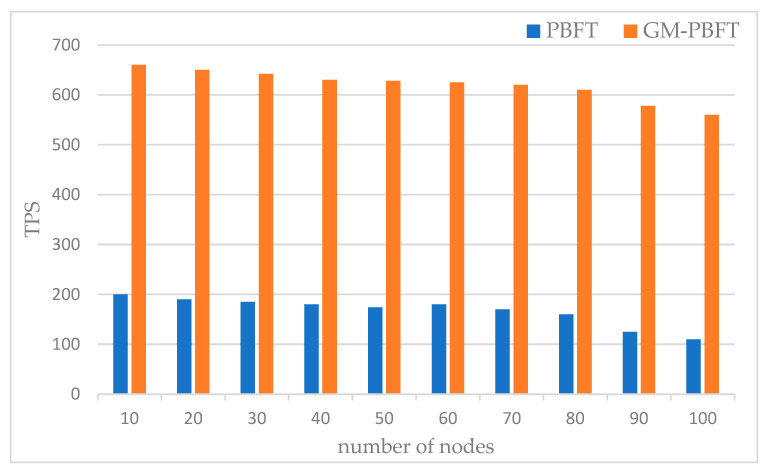
Comparison of the impact of node count on throughput.

The GM-PBFT algorithm and the PBFT method were tested on multiple occasions in an environment with various numbers of nodes to assess transaction throughput, and the average value of the experimental data was taken as the test results. The average time to complete 1000 transactions of the two algorithms was recorded, and the transaction throughput was calculated by the generation time of each block and the number of transactions in the block. Figure 10 shows the comparison of the impact of the number of nodes on throughput.

From the figure, it is known that the throughput of consensus algorithms decreases gradually with the increase in the number of nodes. The TPS of the GM-PBFT algorithm is much larger than the PBFT consensus algorithm with the same size of nodes. The throughput of the blockchain system increases because of the grouped multilayer consensus of the GM-PBFT algorithm. With the increasing size of nodes in the system, the transaction volume will increase, and the throughput of the two algorithms will gradually decrease due to the influence of the upper limit of communication bandwidth. The throughput of the GM-PBFT algorithm is always larger than that of the PBFT algorithm in the whole consensus process, which can handle more transactions per unit of time and is suitable for the environment of a coalition chain with high throughput requirements.

### 4.5. Fault Tolerance Analysis

In terms of fault tolerance, the upper layer consensus phase of the GM-PBFT algorithm is guaranteed by the PBFT consensus algorithm, in which the messages of pre-prepare, prepare, and commit need to be completed in the same view. All messages need to conform to the PBFT consensus process for digest, sequence number, and verification of signatures. The consumption time is shorter than the node-triggered view switching time. The last layer consensus phase adopts the Raft algorithm and the supervisory node row supervisory leader, which makes the in-group consensus resilient to malicious Byzantine behavior.

The security of the GM-PBFT algorithm’s end layer consensus is guaranteed by the supervisory nodes, which makes the consensus resistant to malicious Byzantine behavior and enhances the security of the end Raft consensus. Ordinary nodes only submit log messages to the chain when they receive a commit message from the N-1 layer leader and verify that the message turns out to be true. The traditional PBFT algorithm has a fault tolerance of N/3, but with the GM-PBFT algorithm with the addition of the Raft algorithm and the supervisory nodes, the fault tolerance will be higher than N/3. Therefore, the GM-PBFT algorithm improves the fault tolerance of the system consensus, and there is an improvement in the security of the system. A comparison of the performance of this optimized algorithm with other commonly used consensus algorithms was made and the results are shown in Table 1.

## 5. Conclusions and Outlook

The conventional PBFT algorithm struggles with excessive communication complexity and inadequate transaction scenario performance. The communication difficulty between nodes climbs linearly as the number of nodes in the blockchain system gradually increases, owing to high communication costs. The GM-PBFT consensus algorithm is designed in terms of node grouping strategy, supervision strategy, and grouping multilayer consensus, respectively. The experimental results show that the GM-PBFT algorithm has fewer consensus communications and a shorter consensus delay than the PBFT method, which has a greater throughput and is better suited to blockchain environments with a large number of network nodes. It increases the system’s security and performance index without affecting its fault tolerance. The experiment shows that the GM-PBFT consensus algorithm meets the requirements of the application scenarios of digital asset trading.

The technique is still in the experimental stage, and future research will enhance the GM-PBFT algorithm’s performance to improve system consensus. The optimization method in this paper does not address the limitation of the PBFT algorithm in terms of the number of consensus nodes, and we will focus our research on solving this problem in future work. In order to promote the continual development of blockchain technology, it is believed that the addition of a polycentric multilayer hierarchical structure to the multilayer grouping will further improve the consensus efficiency of blockchain under large-scale nodes. It will also help in choosing more suitable solutions based on the actual results of various blockchain applications combined with the enhanced PBFT algorithm.

## Figures and Tables

**Table 1:** Performance comparison of common consensus algorithms.

Consensus Algorithm	Depletion of Resources	Consensus Efficiency	Degree of Decentralization	Fault Tolerance Rate
Pow	high	low	high	50%
Pos	relatively high	low	high	50%
PBFT	high	relatively high	low	33%
GM-PBFT	relatively high	high	relatively high	>33%

## Data Availability

The data used to support the findings of this study are included within the article.
